# Stochastic Dynamics of a Time-Delayed Ecosystem Driven by Poisson White Noise Excitation

**DOI:** 10.3390/e20020143

**Published:** 2018-02-23

**Authors:** Wantao Jia, Yong Xu, Dongxi Li

**Affiliations:** 1 Department of Applied Mathematics, Northwestern Polytechnical University, Xi’an 710072, China; 2 College of Big Data Science, Taiyuan University of Technology, Taiyuan 030024, China

**Keywords:** predator-prey ecosystem, time delay, stochastic averaging, Poisson white noise

## Abstract

We investigate the stochastic dynamics of a prey-predator type ecosystem with time delay and the discrete random environmental fluctuations. In this model, the delay effect is represented by a time delay parameter and the effect of the environmental randomness is modeled as Poisson white noise. The stochastic averaging method and the perturbation method are applied to calculate the approximate stationary probability density functions for both predator and prey populations. The influences of system parameters and the Poisson white noises are investigated in detail based on the approximate stationary probability density functions. It is found that, increasing time delay parameter as well as the mean arrival rate and the variance of the amplitude of the Poisson white noise will enhance the fluctuations of the prey and predator population. While the larger value of self-competition parameter will reduce the fluctuation of the system. Furthermore, the results from Monte Carlo simulation are also obtained to show the effectiveness of the results from averaging method.

## 1. Introduction

In the real-world ecosystem, a time delay effect in the ecosystems is inevitable. Such as, in the predator-prey type ecosystem, it takes time for the predator population to adjust any change of the prey population. Thus, the ecosystems with time delay effects have received more and more attentions in past decades. Several types of models considering the time delay effects are introduced. The dynamical properties of the ecosystem with time delay, such as stability and bifurcations, have been studied [1,2,3,4,5,6,7]. It indeed can be found that the effect of the time delay can change the behavior of the ecosystem substantially. However, the models in all these studies are deterministic. They are too idealistic since the changes in the environment are not taken into consideration. As a matter of fact, random perturbation is universal in the real world. Thus, the stochastic excitations describing the fluctuations in the environment are added to the deterministic models. The dynamics of the ecosystem under stochastic excitations have been widely investigated [8,9,10,11,12,13,14,15,16,17,18,19,20]. Especially, the stochastic dynamics of the noise-induced predator-prey type ecosystems with time delay, including stochastic responses [9,19,21,22], stochastic stability [23], stochastic resonance [16,24] and noise-delayed extinction [16], have been investigated by some numerical and analytical methods.

It can be noted that the random perturbations in the environment in the previously mentioned works are usually modelled as continuous stochastic processes, such as the Gaussian white noise or Gaussian colored noise. However, the perturbations in the real-world environment are complicated. There are some unavoidable sparse, drastic changes in the environment, for instance sudden natural disasters, earthquakes, forest fires, floods [25,26,27]. These changes are pulse-type perturbations, which cannot be characterized by the continuous stochastic processes properly. Therefore, the stochastic jump processes, such as Poisson white noise, are considered to model such pulse-type perturbations [28,29]. So far, studying the stochastic dynamics of ecosystem with stochastic jump process has attracted more and more attention [30,31,32,33,34]. For example, Zhu and his coauthors have investigated the Lotka–Volterra (LV) system under Poisson white noise by using the generalized cell mapping method [32] and the stochastic averaging method [33]. Duan and Xu have studied the stochastic stability of a logistic model subjected to Poisson white noise process via the Lyapunov exponent [34]. People can find that the influence of Poisson white noise on the ecosystem is different from those of continuous stochastic processes on ecosystem. However, the ecosystems studied in these works are all systems without time delay effect. Consequently, it is necessary to study the stochastic dynamics of time-delayed ecosystems driven by stochastic jump processes.

Inspired by this, we study the stochastic behaviors of a time-delayed predator-prey ecosystem model under discrete random environmental fluctuations. The fluctuations are modeled as Poisson white noises. Due to the existence of the Poisson white noises, it is difficult to obtain the probability density functions (PDFs) of the species populations since the related dynamical equations contain infinite terms. Thus, it is necessary to develop some methods to study this problem. In the present paper, a procedure to calculate the stationary PDFs is formatted theoretically. The stochastic averaging procedure is first applied to deduce the averaged generalized Fokker-Planck-Kolmogorov (GFPK) equation governing the PDF of the first integral. Then, the stationary PDFs of prey and predator populations are obtained by using a perturbation method. Finally, the influences of the time delay, self-competition parameter and the Poisson white noises on the stochastic behaviors of the ecosystem are discussed based on the stationary PDFs. In addition, the results from Monte Carlo simulation are also carried out to show the effectiveness of proposed method.

## 2. Delayed-Type Predator-Prey System with Poisson White Noises

### 2.1. The Deterministic Model with Time Delay Terms

Without considering the random fluctuations in the environment, the time-delayed predator-prey type ecosystem can be described by following integral-differential equation [9]:

(1) $$\begin{array}{l} {\dot{x}}_{1}=a_{1}x_{1}-sx_{1}^{2}-bx_{1}x_{2},\\ {\dot{x}}_{2}=-cx_{2}+fx_{2}\int_{-\infty }^{t}F\left(t-\tau \right)x_{1}\left(\tau \right)d\tau . \end{array}$$

The second equation of Equation (1) can also be written as following equivalent form [3]

(2) $${\dot{x}}_{2}=-cx_{2}+fx_{2}\int_{0}^{\infty }F\left(\tau \right)x_{1}\left(t-\tau \right)d\tau .$$

where $x_{1}$ and $x_{2}$ are the population densities of prey and predator, respectively. The parameters $a_{1},s,b,c$ and f are positive constants. $a_{1}$ is the birth rate of the preys. c is the death rate of the predators. The term $-sx_{1}^{2}$ denotes the prey self-competition. The terms $-bx_{1}x_{2}$ and $fx_{2}\int_{-\infty }^{t}F\left(t-\tau \right)x_{1}\left(\tau \right)d\tau$ provide a balance between the prey and predator populations. The integral term represents the time delay effect of the prey population on the predator population. It means that the change of the prey population affects the predator population after a time lag. And this time delay depends on an average over past populations, not only on the population at some particular instant in the past. F(·) in the integrand is called the delay function and normalized as follows:

$$\int_{0}^{\infty }F\left(t\right)dt=1.$$

Macdonald has presented two ways to reduce the system (1) in [3]. The first one is exact, but, it depends on the choice of a simple form of the time delay function F(·). The other one does not need to specify the time delay function, but needs its moments, i.e.:

(3) $$\gamma =\int_{0}^{\infty }\tau F\left(\tau \right)d\tau$$

Equation (3) can be viewed as a measure of the average delay time. Two reasonable choices for F(t) provieded by Cai and Lin [9] are:

$$F\left(t\right)=\frac{1}{\gamma }e^{-t/\gamma }\mathrm{and}F\left(t\right)=\frac{4}{\gamma^{2}}te^{-2t/\gamma }.$$

To avoid specifying time delay function, the second way is used to reduce the system (1) in the present paper. As pointed out in [3], this method is valid for small γ. Substituting the first-order approximation of the Taylor expansion of $x_{1}\left(t-\tau \right)$ about τ=0 into Equation (2) and applying Equation (3), the first-order approximation of Equation (2) can be derived as:

(4) $${\dot{x}}_{2}=-cx_{2}+fx_{1}x_{2}-f\gamma x_{2}{\dot{x}}_{1}$$

Together with the first equation of Equation (1), the original Equation (1) can be rewritten as:

(5) $$\begin{array}{l} {\dot{x}}_{1}=a_{1}x_{1}-sx_{1}^{2}-bx_{1}x_{2}\\ {\dot{x}}_{2}=-cx_{2}+f\left(1-\gamma a_{1}\right)x_{1}x_{2}+sf\gamma x_{1}^{2}x_{2}+bf\gamma x_{1}x_{2}^{2} \end{array}$$

Let $a=a_{1}-sc/f$. Then the system can be converted to:

(6) $$\begin{array}{l} {\dot{x}}_{1}=x_{1}[a-bx_{2}-\frac{s}{f}\left(fx_{1}-c\right)]\\ {\dot{x}}_{2}=x_{2}[\left(-c+fx_{1}\right)\left(1+s\gamma x_{1}\right)+f\gamma x_{1}\left(bx_{2}-a\right)] \end{array}$$

The system (6) can be used to investigate the effect of the time delay on the populations of the prey and predator. It is easy to check that system (6) has the same equilibrium point $x_{10}=c/f$, $x_{20}=a/b$ as that of the Lotka–Volterra model without time-delay and stochastic excitations [8,9]. In addition, it is pointed in Cai and Lin’s work [9] that, for a physically meaningful and stable ecological system, the choice of the parameters must make the ecosystem stable. All the parameters in the present paper meet this requirement.

### 2.2. Stochastic Model

In the present paper, we suppose that there are some unavoidable sparse, drastic changes in the environment. These changes are modeled as the Poisson white noises. They will cause random jump in the prey growth rate and the predator death rate. Thus, the stochastic model has following form:

(7) $$\begin{array}{l} {\dot{X}}_{1}=X_{1}\left[a-bX_{2}-\frac{s}{f}\left(fX_{1}-c\right)\right]+X_{1}W_{1}\left(t\right)\\ {\dot{X}}_{2}=X_{2}\left[\left(-c+fX_{1}\right)\left(1+s\gamma X_{1}\right)+f\gamma X_{1}\left(bX_{2}-a\right)\right]+X_{2}W_{2}\left(t\right) \end{array}$$

where $W_{1}\left(t\right)$ and $W_{2}\left(t\right)$ are two independent Poisson white noises, which can be defined as [35,36]

(8) $$W_{i}\left(t\right)=\sum_{k=1}^{N_{i}\left(t\right)}Y_{ik}\delta \left(t-t_{ik}\right),\;i=1,2$$

where the δ(•) is the Dirac delta function; $N_{i}\left(t\right)$ is a Poisson counting process with mean arrival rate $\lambda_{i}>0$ and gives the number of the pulses arriving in the time interval [0,t]; $Y_{ik}$ represents the amplitude of the Poisson white noises, which is independent of the pulse arrival time $t_{ik}$. In the present paper, $Y_{ik}$ is Gaussian distribution with mean value 0 and variance $E[Y_{i}^{2}]$ [32,33].

It is hard to obtain the exact solution of Equation (7) analytically, even for the Gaussian white noise case. Therefore, some methods have been developed to solve this problem. Among which, the stochastic averaging method is an analytical efficient method. It has been successfully applied to analyze the stochastic behaviors of the ecosystem under stochastic continuous excitations [18,19,22,37]. In recent years, the stochastic averaging method has been generalized to investigate the nonlinear system with random jump excitations [38,39,40,41]. In the following section, we will employ the stochastic averaging method to study stochastic dynamics of the ecosystem (7).

To proceed further analysis, the following assumptions about the system are in order. First, the coefficient s in self-competition term is generally small. It means that the self-competition term has small influence when the prey population density is small. Second, the parameter γ is small, corresponding to shorter time-delay effect. Finally, the noise intensities of *W*_1_(*t*) and *W*_2_(*t*) are small, indicating small random fluctuations in the system dynamics. These assumptions are generally valid for the real-world ecosystem [8,9]. For the convenience of the further theoretical analysis, in the following part, the parameter s, γ and $\lambda_{i}E[Y_{i}^{2}]$ in Equation (7) are replaced by: $\varepsilon^{2}s$, $\varepsilon^{2}\gamma$ and $\lambda_{i}\varepsilon^{2}E[Y_{i}^{2}]$, respectively, where ε is a small parameter. Thus, Equation (7) can be rewritten as:

(9) $$\begin{array}{l} {\dot{X}}_{1}=X_{1}\left[a-bX_{2}-\frac{\varepsilon^{2}s}{f}\left(fX_{1}-c\right)\right]+\varepsilon X_{1}W_{1}\left(t\right)\\ {\dot{X}}_{2}=X_{2}\left[\left(-c+fX_{1}\right)\left(1+\varepsilon^{4}s\gamma X_{1}\right)+\varepsilon^{2}f\gamma X_{1}\left(bX_{2}-a\right)\right]+\varepsilon X_{2}W_{2}\left(t\right) \end{array}$$

Equation (9) is usually modeled as the Stratonovich stochastic differential equation (SDE) [9,13,18]. By adding some correction terms [13,35,36], it can be converted to following equivalent Itô SDE:

(10) $$\begin{array}{l} dX_{1}=X_{1}\left[a-bX_{2}-\frac{\varepsilon^{2}s}{f}\left(fX_{1}-c\right)\right]dt+X_{1}dC_{1}\left(t\right)+X_{1}\sum_{i=1}^{\infty }\frac{1}{i!}{\left(\varepsilon dC_{1}\left(t\right)\right)}^{i}\\ dX_{2}=X_{2}\left[\left(-c+fX_{1}\right)\left(1+\varepsilon^{4}s\gamma X_{1}\right)+\varepsilon^{2}f\gamma X_{1}\left(bX_{2}-a\right)\right]dt+X_{2}\sum_{i=1}^{\infty }\frac{1}{i!}{\left(\varepsilon dC_{2}\left(t\right)\right)}^{i} \end{array}$$

The terms $X_{1}\sum_{i=2}^{\infty }\frac{1}{i!}{\left(\varepsilon dC_{1}\left(t\right)\right)}^{i}$ and $X_{2}\sum_{i=2}^{\infty }\frac{1}{i!}{\left(\varepsilon dC_{2}\left(t\right)\right)}^{i}$ in Equation (10) are the correction terms from Stratonovich SDE to Itô SDE. Then, Equation (10) can be converted to following equivalent stochastic integro-differential equation (SIDE) [42]:

(11) $$\begin{array}{l} dX_{1}=X_{1}\left[a-bX_{2}-\frac{\varepsilon^{2}s}{f}\left(fX_{1}-c\right)\right]dt+\int_{Y_{1}}h_{11}{𝒫}_{1}\left(dt,dY_{1}\right)\\ dX_{2}=X_{2}[\left(-c+fX_{1}\right)\left(1+\varepsilon^{4}s\gamma X_{1}\right)+\varepsilon^{2}f\gamma X_{1}\left(bX_{2}-a\right)]dt+\int_{Y_{2}}h_{22}{𝒫}_{2}\left(dt,dY_{2}\right) \end{array}$$

in which:

(12) $$h_{11}=X_{1}\sum_{i=1}^{\infty }\frac{\varepsilon^{i}}{i!}{\left(Y_{1}\right)}^{i};h_{22}=X_{2}\sum_{i=1}^{\infty }\frac{\varepsilon^{i}}{i!}{\left(Y_{2}\right)}^{i}$$

and ${𝒫}_{1}\left(dt,dY_{1}\right)$ (i=1,2) are the Poisson random measures; $Y_{i}$(i=1,2) are Poisson mark spaces [42].

### 2.3. Stochastic Averaging Approach

In this subsection, a stochastic averaging procedure is derived to simplify the original system. For future use, we choose the following deterministic conservative system for the stochastic averaging [9,18]:

(13) $$\begin{array}{l} {\dot{x}}_{1}=x_{1}\left(a-bx_{2}\right)\\ {\dot{x}}_{2}=x_{2}\left(-c+fx_{1}\right)\left(1+\varepsilon^{4}s\gamma x_{1}\right) \end{array}$$

The system (13) has the same equilibrium point as that of system (9) without the Poisson white noises. System (13) possesses a first integral [9]

(14) $$r\left(x_{1},x_{2}\right)=fx_{1}-c-c\ln\left(\frac{fx_{1}}{c}\right)+bx_{2}-a-a\ln\left(\frac{bx_{2}}{a}\right)-\varepsilon^{4}cs\gamma x_{1}+\frac{\varepsilon^{4}}{2}fs\gamma x_{1}^{2}+\frac{\varepsilon^{4}c^{2}s\gamma }{2f}$$

**Figure 1:**
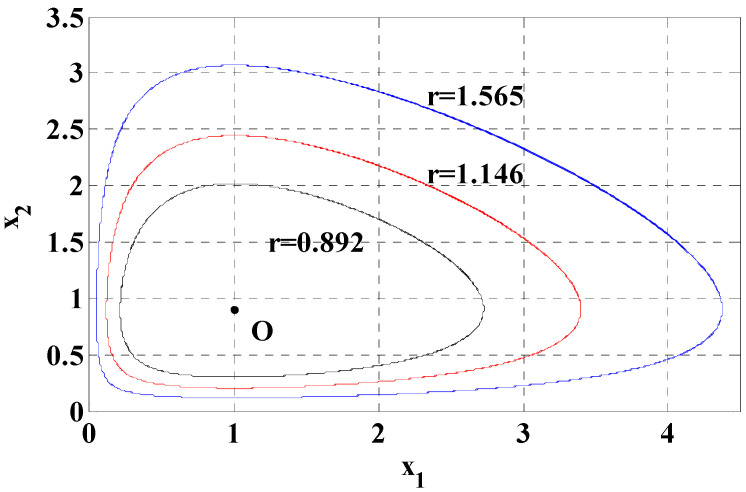
Equilibrium and periodic trajectories of the deterministic system (13).

This first integral can be view as an extension of first integral for the standard LV system [8,43]. It can be seen that $r\left(x_{1},x_{2}\right)=0$ at the equilibrium point (c/f,a/b). And for each positive constant K, $r\left(x_{1},x_{2}\right)=K$ represents a periodic trajectory surrounding the equilibrium point. For illustrative purpose, Figure 1 shows the equilibrium point O and three different periodic trajectories for system (13) with ε=0.1, a=0.9, b=1.0, c=0.5, f=0.5, $\varepsilon^{2}s=0.2$, $\varepsilon^{2}\gamma =0.2$. In this figure, the equilibrium point corresponds to K=0, and the trajectories correspond to K=0.892, 1.146,1.565. And the period of the trajectory can be determined from

(15) $$T\left(K\right)=\oint_{K}dt=\oint_{K}\frac{dx_{2}}{x_{2}\left(-c+fx_{1}\right)\left(1+\varepsilon^{4}s\gamma x_{1}\right)}=\oint_{K}\frac{dx_{1}}{x_{1}\left(a-bx_{2}\right)}$$

in which the integration symbol means integration along the trajectory $r\left(x_{1},x_{2}\right)=K$.

Replace $x_{1}$ and $x_{2}$ by the stochastic processes $X_{1}\left(t\right)$ and $X_{2}\left(t\right)$, the stochastic counterpart of Equation (14) $R\left(t\right)=R\left(X_{1}\left(t\right),X_{2}\left(t\right)\right)$ can be given as:

(16) $$R\left(t\right)=fX_{1}-c-c\ln\left(\frac{fX_{1}}{c}\right)+bX_{2}-a-a\ln\left(\frac{bX_{2}}{a}\right)-\varepsilon^{4}cs\gamma X_{1}+\frac{\varepsilon^{4}}{2}fs\gamma X_{1}^{2}+\frac{\varepsilon^{4}c^{2}s\gamma }{2f}$$

Based on the stochastic jump-diffusion chain rule [42] and Equation (11), the SIDE for R(t) is:

(17) $$\begin{array}{ll} dR= & \left[-\frac{\varepsilon^{2}s}{f}{\left(fX_{1}-c\right)}^{2}\left(1+\varepsilon^{4}s\gamma X_{1}\right)+\varepsilon^{2}f\gamma X_{1}{\left(bX_{2}-a\right)}^{2}\right]dt\\ & +\int_{Y_{1}}\left\{\left(f-\varepsilon^{4}cs\gamma \right)h_{11}+\frac{\varepsilon^{4}}{2}fs\gamma \left(2X_{1}h_{11}+\gamma_{11}^{2}\right)-c\ln\left(1+\frac{h_{11}}{X_{1}}\right)\right\}{𝒫}_{1}\left(dt,dY_{1}\right)\\ & +\int_{Y_{2}}\left\{bh_{22}-a\ln(1+\frac{h_{22}}{X_{2}})\right\}{𝒫}_{2}\left(dt,dY_{2}\right) \end{array}$$

In order to possess the stochastic averaging method, consider the Taylor expansion of $\ln\left(1+h_{11}/X_{1}\right)$ and $\ln\left(1+h_{22}/X_{2}\right)$ in Equation (17), and then substitute Equation (12) to Equation (17). After collecting the terms of same order of ε, Equation (17) can be written as:

(18) $$\begin{array}{ll} dR= & \left[-\frac{\varepsilon^{4}s}{f}{\left(fX_{1}-c\right)}^{2}\left(1+\varepsilon^{4}s\gamma X_{1}\right)+\varepsilon^{2}f\gamma X_{1}{\left(bX_{2}-a\right)}^{2}\right]dt\\ & +\int_{Y_{1}}\left[\varepsilon A_{11}Y_{1}+\varepsilon^{2}A_{12}Y_{1}^{2}+\varepsilon^{3}A_{13}Y_{1}^{3}+\varepsilon^{4}A_{14}Y_{1}^{4}+\cdots \right]{𝒫}_{1}\left(dt,dY_{1}\right)\\ & +\int_{Y_{2}}\left[\varepsilon A_{21}Y_{2}+\varepsilon^{2}A_{22}Y_{2}^{2}+\varepsilon^{3}A_{23}Y_{2}^{3}+\varepsilon^{4}A_{24}Y_{2}^{4}+\cdots \right]{𝒫}_{2}\left(dt,dY_{2}\right) \end{array}$$

where:

(19) $$A_{11}=\left(fX_{1}-c\right)\left(\varepsilon^{4}s\gamma X_{1}+1\right);$$

(20) $$A_{12}=\frac{1}{2}\left[\left(fX_{1}-c\right)\left(\varepsilon^{4}s\gamma X_{1}+1\right)+\left(\varepsilon^{4}fs\gamma X_{1}^{2}+c\right)\right];$$

(21) $$A_{13}=\left[\frac{1}{6}\left(fX_{1}-c\right)\left(\varepsilon^{4}s\gamma X_{1}+1\right)+\frac{1}{2}\left(\varepsilon^{4}fs\gamma X_{1}^{2}+c\right)-\frac{c}{3}\right];$$

(22) $$A_{14}=\left[\frac{1}{24}\left(fX_{1}-c\right)\left(\varepsilon^{4}s\gamma X_{1}+1\right)+\frac{7}{24}\left(\varepsilon^{4}fs\gamma X_{1}^{2}+c\right)-\frac{c}{4}\right];$$

(23) $$A_{21}=\left(bX_{2}-a\right);A_{22}=\frac{1}{2}\left[\left(bX_{2}-a\right)+a\right];$$

(24) $$A_{23}=\frac{1}{6}\left[\left(bX_{2}-a\right)+a\right];A_{24}=\frac{1}{24}\left[\left(bX_{2}-a\right)+a\right].$$

It can be seen from Equation (18) that *R*(*t*) is a slowly varying stochastic process since ε is a small parameter. According to the stochastic averaging method for the nonlinear system under the Poisson white noise [33], the averaged GFPK equation can be derived as:

(25) $$\begin{array}{ll} \frac{\partial }{\partial t}p\left(r,t\right)= & -\frac{\partial }{\partial r}\left(\left(\varepsilon^{2}{\bar{A}}_{11}\left(r\right)+\varepsilon^{4}{\bar{A}}_{12}\left(r\right)\right)p\left(r,t\right)\right)+\frac{1}{2!}\frac{\partial^{2}}{\partial r^{2}}\left(\left(\varepsilon^{2}{\bar{A}}_{21}\left(r\right)+\varepsilon^{4}{\bar{A}}_{22}\left(r\right)\right)p\left(r,t\right)\right)\\ & -\frac{1}{3!}\frac{\partial^{3}}{\partial r^{3}}\left(\varepsilon^{4}{\bar{A}}_{3}p\left(r,t\right)\right)+\frac{1}{4!}\frac{\partial^{4}}{\partial r^{4}}\left(\varepsilon^{4}{\bar{A}}_{4}p\left(r,t\right)\right)+O\left(\varepsilon^{6}\right) \end{array}$$

where $O\left(\varepsilon^{6}\right)$ contains the term of the order of $\varepsilon^{6}$ and higher, and the coefficients for the GFPK equation are given in Appendix A.

Note that the SIDE (18) contains infinite terms. To get the closed form of averaged equations, truncation is needed during the averaging process. In the averaged GFPK Equation (25), only the terms up to the fourth order of ε are considered for simplicity.

### 2.4. Stationary Probability Density Functions

The averaged GFPK equation can be solved with certain boundary and initial conditions. The stationary PDF of R(t), denoted by p(r), can be obtained by solving the reduced GFPK equation:

(26) $$\begin{array}{ll} 0= & -\frac{\partial }{\partial r}\left(\left(\varepsilon^{2}{\bar{A}}_{11}\left(r\right)+\varepsilon^{4}{\bar{A}}_{12}\left(r\right)\right)p\left(r\right)\right)+\frac{1}{2!}\frac{\partial^{2}}{\partial r^{2}}\left(\left(\varepsilon^{2}{\bar{A}}_{21}\left(r\right)+\varepsilon^{4}{\bar{A}}_{22}\left(r\right)\right)p\left(r\right)\right)\\ & -\frac{1}{3!}\frac{\partial^{3}}{\partial r^{3}}\left(\varepsilon^{4}{\bar{A}}_{3}\left(r\right)p\left(r\right)\right)+\frac{1}{4!}\frac{\partial^{4}}{\partial r^{4}}\left(\varepsilon^{4}{\bar{A}}_{4}\left(r\right)p\left(r\right)\right)+O\left(\varepsilon^{6}\right) \end{array}$$

with the following conditions:

(27) $${p\left(r\right)|}_{r\to \infty }=0\mathrm{and}\int_{0}^{\infty }p\left(r\right)dr=1.$$

The perturbation method [33,44] is used to solve the reduced GFPK equation. The second order perturbation solution $p\left(r\right)=p_{0}\left(r\right)+\varepsilon p_{1}\left(r\right)+\varepsilon^{2}p_{2}\left(r\right)$ is adopted in our calculation. On substituting the second order perturbation solution into Equation (27) and grouping terms of the same order of ε, the following set of differential equations is obtained:

In $\varepsilon^{2}$,

(28) $$-\frac{d}{dr}\left({\bar{A}}_{11}p_{0}\left(r\right)\right)+\frac{1}{2}\frac{d^{2}}{dr^{2}}\left({\bar{A}}_{21}p_{0}\left(r\right)\right)=0$$

In $\varepsilon^{3}$,

(29) $$-\frac{d}{dr}\left(\varepsilon {\bar{A}}_{11}p_{1}\left(r\right)\right)+\frac{1}{2}\frac{d^{2}}{dr^{2}}\left(\varepsilon {\bar{A}}_{21}p_{1}\left(r\right)\right)=0$$

In $\varepsilon^{4}$,

(30) $$-\frac{d}{dr}\left(\varepsilon^{2}{\bar{A}}_{11}p_{2}\left(r\right)\right)+\frac{1}{2}\frac{d^{2}}{dr^{2}}\left(\varepsilon^{2}{\bar{A}}_{21}p_{2}\left(r\right)\right)=\frac{1}{3!}\frac{d^{3}}{dr^{3}}\left({\bar{A}}_{3}p_{0}\left(r\right)\right)-\frac{1}{4!}\frac{d^{4}}{dr^{4}}\left({\bar{A}}_{4}p_{0}\left(r\right)\right)$$

Equation (28) is a Fokker-Planck-Kolmogorov equation for Gaussian white noise excitation. The exact solution can be obtained if the FPK Equation (28) belongs to the class of generalized stationary potential [39]. The perturbation solutions $p_{0}\left(r\right)$, $p_{1}\left(r\right)$ and $p_{2}\left(r\right)$ can be obtained by solving Equations (28)–(30) successively.

After getting p(r), the stationary PDFs of $X_{1}$ and $X_{2}$ can be calculated as follows [9,13]:

(31) $$p_{X_{1}X_{2}}\left(x_{1},x_{2}\right)=\frac{p\left(r\right)}{x_{1}x_{2}T\left(r\right)},p_{X_{1}}\left(x_{1}\right)=\int_{0}^{\infty }p_{X_{1}X_{2}}\left(x_{1},x_{2}\right)dx_{2},\;p_{X_{2}}\left(x_{2}\right)=\int_{0}^{\infty }p_{X_{1}X_{2}}\left(x_{1},x_{2}\right)dx_{1}$$

where r is the function of $x_{1}$ and $x_{2}$ given in Equation (14) and T(r) is the quasi-period with the form in Equation (15). The other statics can be easily derived from Equation (31).

## 3. Results

**Figure 2:**
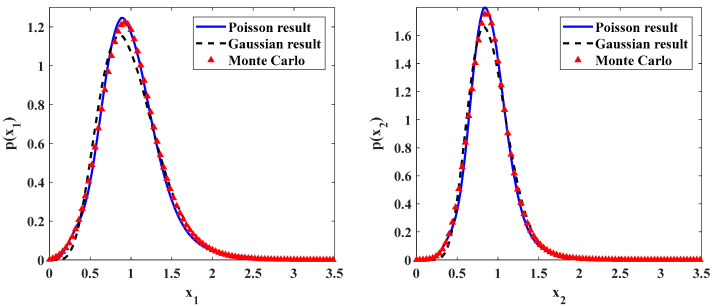
Probability density functions of prey and predator populations for parameters: ε=0.1, a=0.9, b=1.0, f=0.5, c=0.5, $\varepsilon^{2}s=0.2$, $\varepsilon^{2}\gamma =0.1$, $\lambda_{1}=\lambda_{2}=0.3$, $\lambda_{1}\varepsilon^{2}E[Y_{1}^{2}]=\lambda_{2}\varepsilon^{2}E[Y_{2}^{2}]=0.015$.

**Figure 3:**
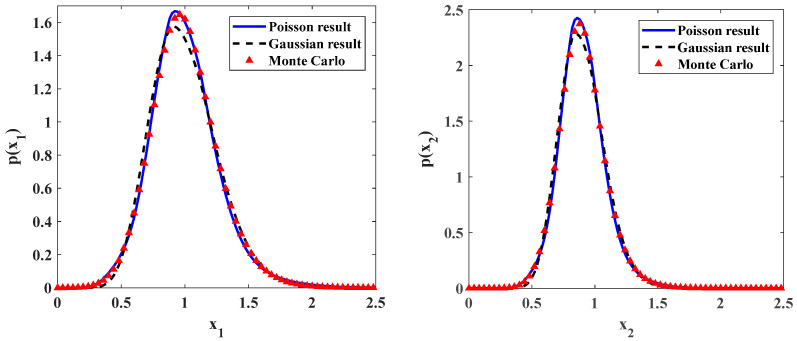
Probability density functions of prey and predator populations for parameters: ε=0.1, a=0.9, b=1.0, f=0.5, c=0.5, $\varepsilon^{2}s=0.2$, $\varepsilon^{2}\gamma =0.1$, $\lambda_{1}=\lambda_{2}=0.4$, $\lambda_{1}\varepsilon^{2}E[Y_{1}^{2}]=\lambda_{2}\varepsilon^{2}E[Y_{2}^{2}]=0.0075$.

Some numerical results are presented in Figure 2 and Figure 3 for ecosystem (7) with different noise intensities. The parameters are shown in the captions of these figures. In these figures, the solid lines are the results of ecosystem under Poisson white noises obtained by the proposed method. The dotted lines are the results from Monte Carlo simulation. A good agreement between the theoretical and simulation results in both figures shows the accuracy of the proposed method. For the purpose of comparison, the stationary PDFs for the time-delayed ecosystem under Gaussian white noises with the same intensity are also depicted in these figures. They are denoted by the dashed lines. One can see that the approximate stationary PDFs for time-delayed ecosystem under Poisson white noise are higher than those for system under Gaussian white noise. It means that, for the same noise intensity, the influence of the Gaussian white noises on the ecosystem is stronger than that of the Poisson white noises.

In the Monte Carlo simulation for ecosystem (7), the Runge-Kutta method for the Poisson white noise excitation proposed by Di Paola and the triangular pulse model of the Poisson white noise excitation are adopted [35]. It is important to note that the numerical simulation time should be long enough to get the stationary responses. In present paper, for each set of system parameters, eight samples are simulated. The time of Monte Carlo simulation is 400,000,000 units for each sample, and the time step length is 0.001.

Cai and Lin [8] show that the stability of system (6) depends on the prey self-competition parameter and the time-delay parameter. Thus, in the following sections, we will investigate the influences of the parameters $\varepsilon^{2}s$ and $\varepsilon^{2}\gamma$ on the ecosystem (7). The stationary PDFs and the statics of prey and predator populations for different values of these two parameters are given and discussed in following sections.

### 3.1. The Effects of the Time Delay Parameter $\varepsilon^{2}\gamma$

**Figure 4:**
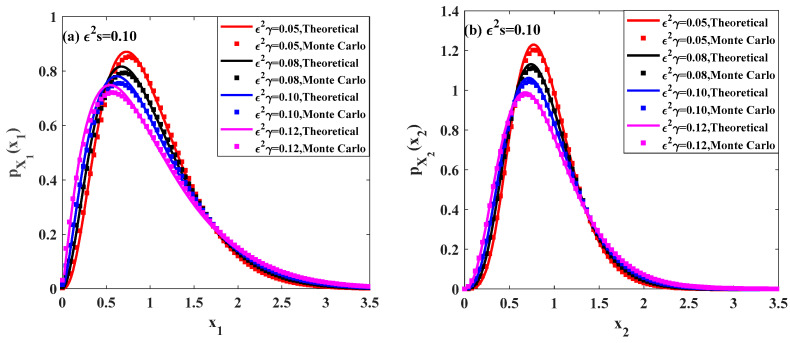
Probability density functions of prey population and predator population for different values of parameter $\varepsilon^{2}\gamma$.

**Figure 5:**
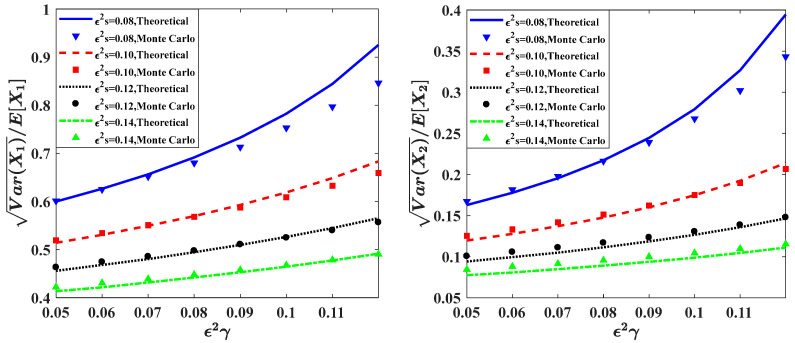
Relative fluctuations versus the time delay parameter $\varepsilon^{2}\gamma$ for different values of parameter $\varepsilon^{2}s$. The other parameters are the same as those in Figure 4.

In this subsection, we investigate the influence of the time delay on the ecosystem (7). Figure 4 and Figure 5 show the effects of the time delay parameter on the PDFs and relative fluctuations $\sqrt{Var\left(X_{i}\right)}/E\left(X_{i}\right)$ of the prey population and predator population.

Figure 4a,b depict the stationary PDFs of the prey population $p_{X_{1}}\left(x_{1}\right)$ and predator population $p_{X_{2}}\left(x_{2}\right)$ for different values of time delay $\varepsilon^{2}\gamma$, respectively. In these figures, the solid lines are the theoretical results obtained by the proposed method. It can be found that, with increasing the time delay $\varepsilon^{2}\gamma$ from 0.05 to 0.12, the peak values of both $p_{X_{1}}\left(x_{1}\right)$ and $p_{X_{2}}\left(x_{2}\right)$ become lower, and the probabilities in both lower and higher population become higher. Besides, the results from Monte Carlo simulation are also plotted to show the effectiveness of the proposed method. The other parameters of the system are given as:ε=0.1, b=1.0, c=0.5, f=0.5, $\lambda_{1}=\lambda_{2}=1.0$, $\varepsilon^{2}\lambda_{1}E[Y_{1}^{2}]=\varepsilon^{2}\lambda_{2}E[Y_{2}^{2}]=0.015$.

Figure 5 shows the relative fluctuations of the prey population and predator population changing versus the values of the time delay parameter $\varepsilon^{2}\gamma$ for different self-completion parameter $\varepsilon^{2}s$. In this figure, the results obtained by the Monte Carlo simulation are represented by point lines, while the results obtained by the proposed method are shown by solid or dashed lines. Each curve of relative fluctuation increases monotonously with the increase of time delay. It means that the fluctuations of the prey population and predator population increase when the time delay is relatively large. Namely, the longer time delay will lead to a wider range distribution of the population, indicating that the ecosystem becomes less stable.

### 3.2. The Effects of the Self-Competition Parameter $\varepsilon^{2}s$

**Figure 6:**
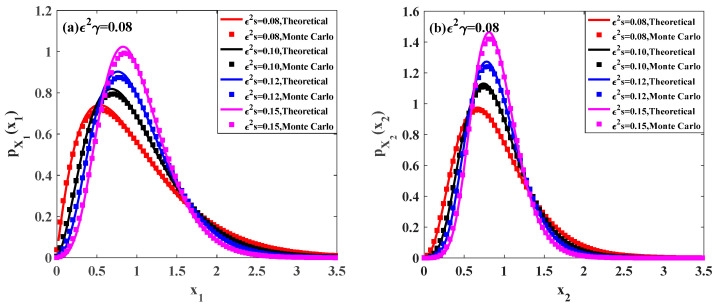
Probability density functions of prey and predator populations for different values of parameter $\varepsilon^{2}s$.

**Figure 7:**
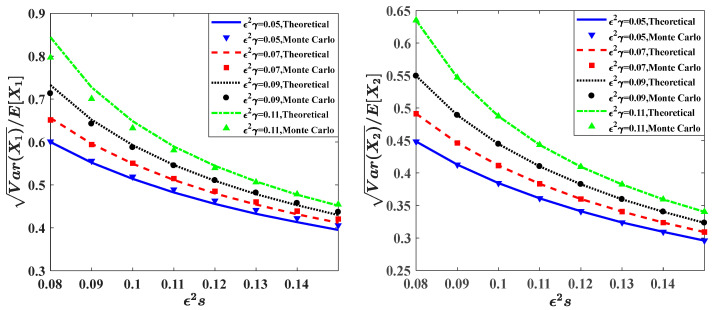
Relative fluctuations versus the time self-competition parameter $\varepsilon^{2}s$ for different values of parameter $\varepsilon^{2}\gamma$. The other parameters are the same as those in Figure 6.

In this subsection, we will investigate the influence of the self-competition parameter $\varepsilon^{2}s$. In Figure 6, the stationary PDFs of prey population and predator population for different values of $\varepsilon^{2}s$ are given. Figure 7 shows the dependence of the relative fluctuations $\sqrt{Var\left(X_{i}\right)}/E\left(X_{i}\right)$ on the self-competition parameter $\varepsilon^{2}s$ for different values of time delay parameter $\varepsilon^{2}\gamma$.

In Figure 6, it can be seen that, for fixed value of other parameters, with the increasing value of $\varepsilon^{2}s$, the PDFs become more concentrated, and the peak value of the PDFs become higher. This implies that the system will become more stable when increasing $\varepsilon^{2}s$ from 0.08 to 0.15. In addition, the results obtained from the Monte Carlo simulation are also calculated to show the validity of the proposed method in the figure. The other system parameters are: ε = 0.1, a=0.9, b=1.0, f=0.5, c=0.5, $\lambda_{1}=\lambda_{2}=1.0$, $\varepsilon^{2}E[Y_{1}^{2}]=\varepsilon E[Y_{2}^{2}]=0.015$.

Figure 7 shows the relative fluctuations of the prey population and the predator population versus the self-competition parameter $\varepsilon^{2}s$ for different values of time delay $\varepsilon^{2}\gamma$. Compared with Figure 5, the trends of the relative fluctuations curves are different. For each value of the time delay parameter $\varepsilon^{2}\gamma$, the curve of the relative fluctuations of both prey population and predator population decrease monotonously with the increase of the self-competition parameter $\varepsilon^{2}s$. It reveals that the fluctuations of the system will decrease when increase $\varepsilon^{2}s$, which indicates that the system becomes more stable for larger $\varepsilon^{2}s$.

### 3.3. The Effects of the Poisson White Noise 

It is known that the mean arrival rate λ and the variance of the noise amplitude $E[Y^{2}]$ are two key parameters for the Poisson white noise. In the following section, the influences of λ and $E[Y^{2}]$ are investigated respectively.

**Figure 8:**
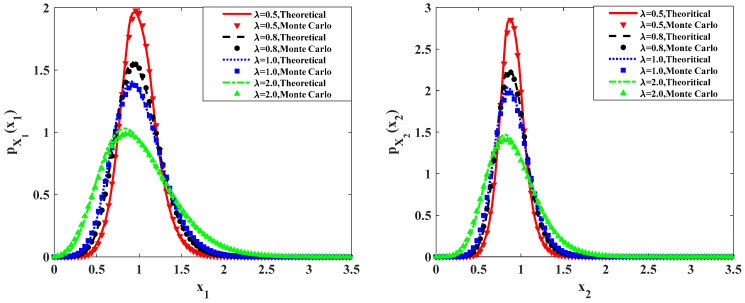
Probability density functions of prey and predator populations for different values of parameter λ.

**Figure 9:**
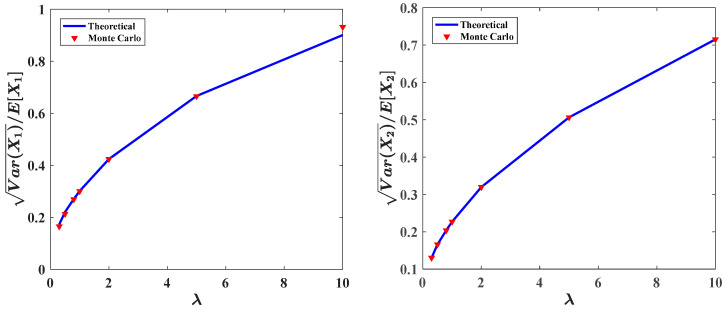
Relative fluctuations of the predator population and prey population for different values of parameter λ.

Figure 8 shows the effects of the mean arrival rate $\lambda_{1}=\lambda_{2}=\lambda$ on the stationary PDFs. The results are calculated with following parameters: ε=0.1, a=0.9, b=1.0, c=0.5, f=0.5, $\varepsilon^{2}s=0.2$, $\varepsilon^{2}\gamma \;=\;0.1$. It can be seen that, when fix the value $\varepsilon^{2}E[Y_{1}^{2}]=\varepsilon^{2}E[Y_{2}^{2}]=\varepsilon^{2}E[Y^{2}]=0.01$, with increasing the mean arrival rate λ from 0.5 to 2.0, the range of the vibration of the predator population and prey population become larger, and the peak values of the PDFs become lower. And the probabilities in both lower and higher population become higher, indicating a less stable system. This is reasonable. A larger mean arrival rate of the Poisson white noise implies more pulses per unit time. The more the number of pulses is, the more unstable the ecosystem will be. The same conclusion can be obtained from Figure 9, which shows the relative fluctuation of the predator population and prey population versus the mean arrival rate λ. As shown in this figure, the curves of the relative fluctuations for both the predator population and prey population increase monotonously as increasing the mean arrival rate λ. This implies that the system has become unstable. The other parameters are the same as those in Figure 8. Also depicted in Figure 8 and Figure 9 are results obtained from the Monte Carlo simulation. 

**Figure 10:**
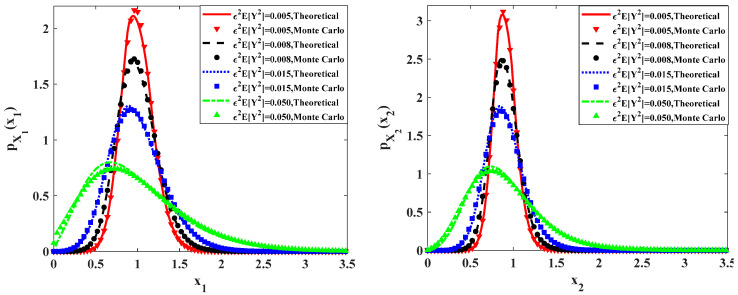
Probability density functions of prey and predator populations for different values of parameter $\varepsilon^{2}E[Y^{2}]\;=\;\varepsilon^{2}E[Y_{1}^{2}]\;=\;\varepsilon^{2}E[Y_{2}^{2}]$.

**Figure 11:**
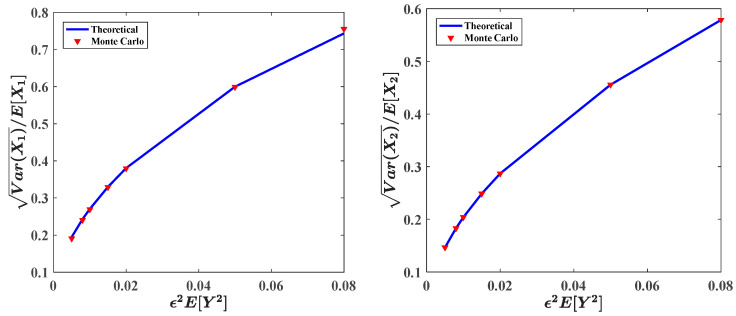
Relative fluctuations of the predator population and prey population for different values of parameter $\varepsilon^{2}E[Y^{2}]\;=\;\varepsilon^{2}E[Y_{1}^{2}]\;=\;\varepsilon^{2}E[Y_{2}^{2}]$.

In Figure 10 and Figure 11, the influences of $\varepsilon^{2}E[Y^{2}]=\varepsilon^{2}E[Y_{1}^{2}]=\varepsilon^{2}E[Y_{2}^{2}]$ on the ecosystem are calculated with system parameters: ε=0.1, a=0.9, b=1.0, c=0.5, f=0.5, $\varepsilon^{2}s=0.2$, $\varepsilon^{2}\gamma =0.1$, $\lambda_{1}=\lambda_{2}=0.8$. Figure 10 and Figure 11 depict the dependence of the stationary PDFs and relative fluctuations of the predator population and prey population. We can arrive at similar conclusions obtained from Figure 8 and Figure 9. As increasing the variance of the impulses $\varepsilon^{2}E[Y^{2}]$, the ecosystem becomes less stable. This agrees with the intuitive consideration. When increase the value of $\varepsilon^{2}E[Y^{2}]$, the fluctuation of the pulses will become larger, which will result in bigger fluctuation of ecosystem. Besides, the results from Monte Carlo simulation represented by dotted lines are also shown in these two figures.

**Figure 12:**
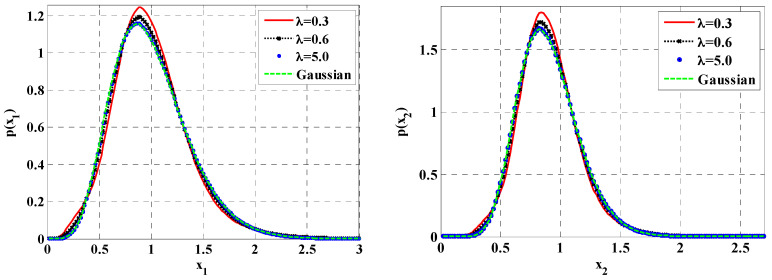
Probability density functions of prey and predator populations for different values of mean arrival rates. The system parameters are ε=0.1, a=0.9, b=1.0, f=0.5, c=0.5, $\varepsilon^{2}s=0.2$, $\varepsilon^{2}\gamma =0.1$.

Shown in Figure 12 are the stationary PDFs of the prey and predator populations for different mean arrival rates of the Poisson white noises, corresponding to the same noise intensity $\lambda_{i}\varepsilon^{2}E[Y_{i}^{2}]$. The results presented in this figure are all obtained by using the proposed method. It is seen that, as increasing the value of the mean arrival rate of the Poisson white noises from 0.3 to 5.0, the approximate stationary PDFs of prey and predator populations of proposed ecosystem under Poisson white noises become closer to those of ecosystem under Gaussian white noises with the same intensity. This implies that the non-Gaussian behavior depends on the mean arrival rate of the Poisson white noise. The other system parameters are given in the caption of this figure.

## 4. Conclusions

This paper investigates the stochastic behaviors of a time-delayed predator-prey ecosystem under pulse-type stochastic environment fluctuations. In this model, the stochastic fluctuations are characterized by the Poisson white noises, and the time delay in the interaction between the prey and predator is described approximately by a time delay parameter. The original ecosystem is first modeled as the Stratonovich SDE and then transferred to Itô SDE. Under some reasonable hypothesises, the stochastic averaging method is applied to simplify the original system. Finally, the approximate stationary PDFs of the prey and predator populations are obtained by using the perturbation method. To verify the accuracy of the proposed method, the results from Monte Carlo simulation are also calculated.

Based on the approximate stationary PDFs of prey and predator populations, the influences of the prey self-competition parameter, the time-delay parameter and the Poisson white noise excitation on the system are investigated in detail. The results show that, increasing time delay parameter as well as the mean arrival rate and the variance of the impulse of the Poisson white noise will enhance the fluctuations of the prey and predator population and make the ecosystem less stable. While the larger value of self-competition parameter will reduce the fluctuations of the system and increase the stability of the ecosystem.

In our present paper, only the effects of some system parameters are studied. Similar approaches may be adopted to study the influences of other system parameters. In the present investigation, the amplitudes of the Poisson white noises are assumed to be Gaussian distribution. They can be other types of distributions depending on the realistic situation.

## Appendix A

The coefficients of GFPK equation are:

$${\bar{A}}_{11}\left(r\right)={\langle \left[-\frac{s}{f}{\left(fX_{1}-c\right)}^{2}+f\gamma X_{1}{\left(bX_{2}-a\right)}^{2}\right]\rangle }_{t}+{\langle \frac{fX_{1}}{2}\rangle }_{t}\lambda_{1}E[Y_{1}{}^{2}]+{\langle \frac{bX_{2}}{2}\rangle }_{t}\lambda_{2}E[Y_{2}{}^{2}];$$

$${\bar{A}}_{12}\left(r\right)={\langle \frac{fX_{1}}{24}\rangle }_{t}\lambda_{1}E[Y_{1}{}^{4}]+{\langle \frac{bX_{2}}{24}\rangle }_{t}\lambda_{2}E[Y_{2}{}^{4}];$$

$${\bar{A}}_{21}\left(r\right)={\langle {\left(fX_{1}-c\right)}^{2}\rangle }_{t}\lambda_{1}E[Y_{1}{}^{2}]+{\langle {\left(bX_{2}-a\right)}^{2}\rangle }_{t}\lambda_{2}E[Y_{2}{}^{2}];$$

$${\bar{A}}_{22}\left(r\right)={\langle \frac{f}{12}X_{1}\left(7fX_{1}-4c\right)\rangle }_{t}\lambda_{1}E[Y_{1}{}^{4}]+{\langle \frac{b}{12}X_{2}\left(7bX_{2}-4a\right)\rangle }_{t}\lambda_{2}E[Y_{2}{}^{4}];$$

$${\bar{A}}_{4}\left(r\right)={\langle \varepsilon^{4}{\left(fX_{1}-c\right)}^{4}\rangle }_{t}\lambda_{1}E[Y_{1}{}^{4}]+{\langle \varepsilon^{4}{\left(bX_{2}-a\right)}^{4}\rangle }_{t}\lambda_{2}E[Y_{2}{}^{4}];$$

where ${\langle \left[\;\right]\rangle }_{t}$ denotes the time average in one quasiperiod, defined as

(A1) $${\langle \left[\;\right]\rangle }_{t}=\frac{1}{T}\oint_{r}\left[\;\right]dt=\frac{1}{T}\oint_{r}\frac{\left[\;\right]dx_{2}}{x_{2}\left(fx_{1}-c\right)\left(1+\varepsilon^{4}s\gamma x_{1}\right)}=\frac{1}{T}\oint_{r}\frac{\left[\;\right]dx_{1}}{x_{1}\left(a-bx_{2}\right)}$$

and T(•) is the period given in Equation (15).
